# Comparison of simultaneous multi-slice readout-segmented EPI and conventional single-shot EPI for diffusion tensor imaging of the ulnar nerve

**DOI:** 10.1016/j.heliyon.2018.e00853

**Published:** 2018-10-16

**Authors:** Michael Ho, Anton Becker, Erika Ulbrich, Andrei Manoliu, Félix P. Kuhn, Matthias Eberhard, Lukas Filli

**Affiliations:** a Institute of Diagnostic and Interventional Radiology, University Hospital Zurich, University of Zurich, Switzerland; b Department of Neuroradiology, University Hospital Freiburg, Freiburg, Germany

**Keywords:** Medical imaging, Neuroscience, Neurology

## Abstract

**Purpose:**

To compare conventional single-shot echo planar imaging (ss-EPI) and simultaneous multi-slice (SMS) readout-segmented EPI (rs-EPI) for magnetic resonance diffusion tensor imaging (DTI) of the ulnar nerve.

**Materials and methods:**

This study was approved by the local ethics committee. Ten healthy volunteers (mean age 30.4 ± 4.01 years; range 25–36 years) underwent 3T DTI of the ulnar nerve at the level of the cubital tunnel. DTI was performed based on ss-EPI as well as SMS rs-EPI sequences. Signal-to-noise ratio (SNR), image quality, and DTI parameters in the ulnar nerve (fractional anisotropy, FA; mean diffusivity, MD) were compared between the two sequences by two independent radiologists.

**Results:**

Acquisition time was 5:12 min for ss-EPI and 5:18 min for SMS rs-EPI. Between the two sequences, no significant differences were found for derived DTI measures FA (p = 0.11) and MD values (p = 0.93). Compared to conventional ss-EPI, SMS rs-EPI yielded significantly less ghosting artifacts (p = 0.04) but inferior nerve depiction (p = 0.001) and worse overall image quality (p = 0.008).

**Conclusion:**

SMS rs-EPI is not advantageous over ss-EPI in DTI of the ulnar nerve at the level of the cubital tunnel.

## 1 Introduction

Ulnar neuropathy at the elbow (UNE) is amongst the most common peripheral neuropathies with an annual incidence of about 21/100.000 [1]. UNE is clinically and socio-economically important since it can lead to continuing atrophy and persistent impairment of intrinsic musculature [2]. Traditional diagnostic testing for UNE mainly consists of physical examination, nerve conduction studies and ancillary neurosonography, all of which are highly operator dependent. Recently, T2-weighted MR neurography has added diagnostic value in precise localization of lesion of UNE [3, 4]. Still, the etiology of ulnar neuropathy at the elbow remains unclear in many cases [1, 5].

In search of additional tools for characterizing peripheral nerves, diffusion tensor imaging (DTI) has been increasingly used in the last years for imaging of forearm nerves [6, 7]. DTI allows voxel-wise measurement of the intensity, main direction, and anisotropy of diffusion of water molecules in body tissues [8]. DTI parameters could have a role as a surrogate marker of peripheral nerve integrity [9, 10]. Furthermore, DTI is projected to play a role in monitoring regeneration and degeneration in motor neuron disease in the future [11]. In particular, the median nerve has been extensively studied [12, 13]. The ulnar nerve, in contrast, has only been subject of investigation in few studies [3, 14, 15].

Conventional DTI is technically based on single-shot echo planar imaging (ss-EPI), where the entire k-space is filled during a single T2* decay. Since ss-EPI is prone to motion artifacts, susceptibility artifacts and image blurring, DTI of peripheral nerves is not yet commonly used in clinical routine. Readout-segmented EPI (rs-EPI) is a modern technique, which may overcome the limitations of ss-EPI, because it achieves shorter echo-spacing by dividing the k-space into separately acquired segments [16]. The main disadvantage of rs-EPI is its longer acquisition time. However, the acquisition time could now be reduced by using the simultaneous multi-slice (SMS) technique with blipped ‘Controlled Aliasing In Parallel Imaging Results In Higher Acceleration’ (blipped CAIPIRINHA). This technique was initially developed for ss-EPI and recently introduced for rs-EPI [17, 18].

In the present work, we tested the feasibility of DTI of the ulnar nerve using SMS rs-EPI. Our hypothesis was that SMS rs-EPI would yield significantly higher image quality compared to conventional ss-EPI at comparable acquisition time.

## 2 Materials and Methods

### 2.1 Study population

The ulnar nerve was scanned in ten healthy volunteers (5 males, 5 females; mean age 30.4; age range 25–36; side, 7 right, 3 left). No subject reported symptoms of peripheral neuropathy or previous cervical spine, upper arm or elbow surgery. Approval by the local ethics committee and written informed consent from all volunteers were obtained.

### 2.2 Imaging protocol

**Fig. 1:**
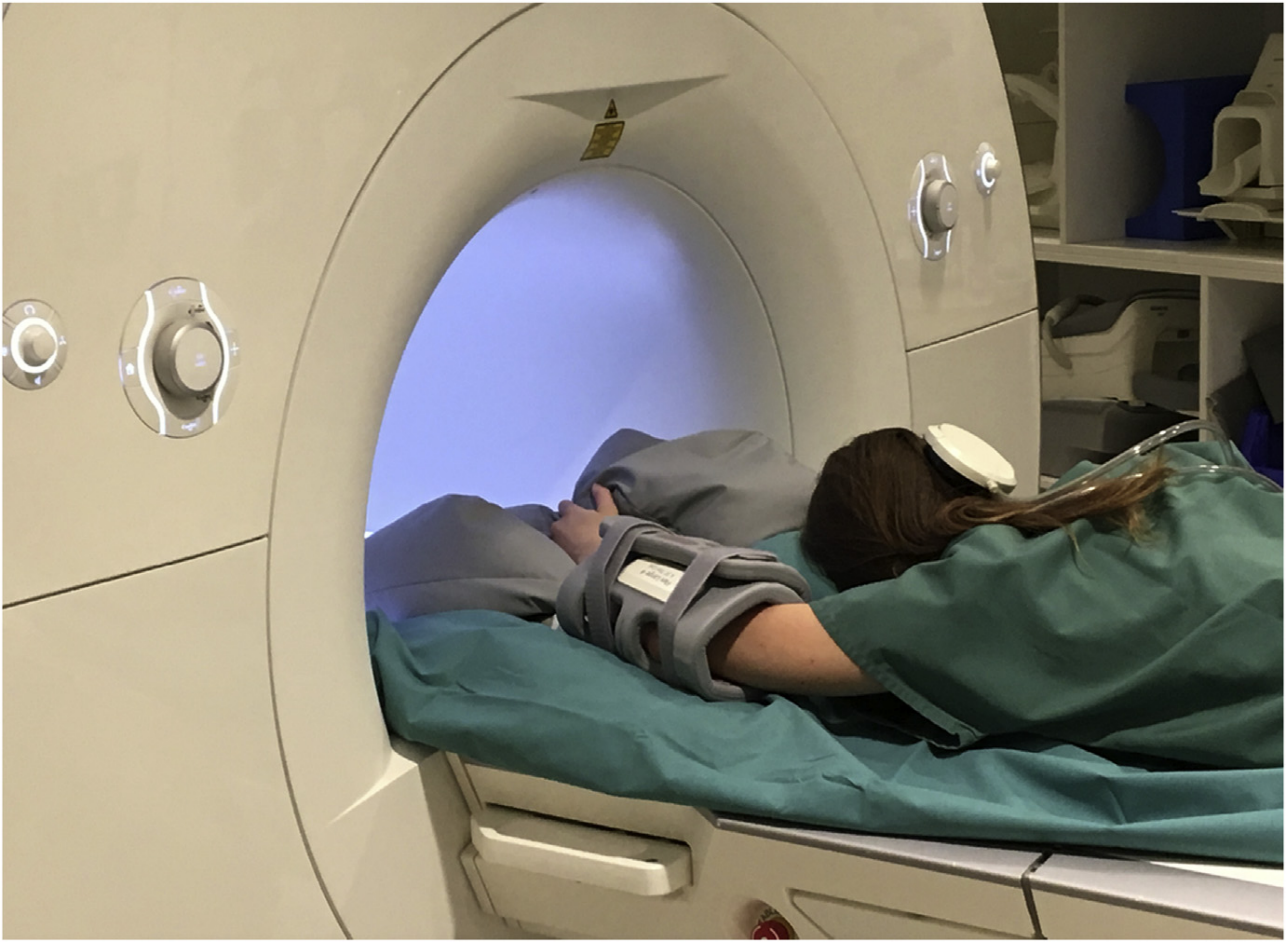
MR scanning positioning: The figure shows a volunteer, who has been positioned in prone position for MR imaging of the ulnar nerve at the level of the elbow - the so called “superman position” - with the arm extended overhead. A 4-channel flexible coil (Siemens Healthcare) is placed around the elbow.

All subjects were scanned in a clinical 3T scanner (MAGNETOM Skyra, Siemens Healthcare, Erlangen, Germany). They were positioned in the prone position with the arm extended overhead. A 4-channel flexible coil (Siemens Healthcare) was placed around the elbow (Fig. 1).

All sequences were planned in the axial plane at the level of the cubital tunnel. First, a T2-weighted fat-suppressed sequence was acquired in order to assess the anatomy and to exclude structural pathologies (TR, 5800 ms; TE, 40 ms; field-of-view, 272 × 320 mm^2^; voxel size, 0.2 × 0.2 × 3.0 mm^3^; slices, 45; slice gap, 0.3 mm; acquisition time, 3:01 min).

Next, DTI was performed using both a conventional ss-EPI sequence and an SMS rs-EPI sequence with two-fold slice acceleration (imaging parameters see Table 1). Acquisition times of both diffusion sequences were almost equaled for reasons of comparison and were kept reasonably short for potential future application in clinical routine.

**Table 1:** Sequence parameters. Abbreviations: ss-EPI, single shot echo planar diffusion-tensor imaging; rs-DTI, readout-segmented echo planar diffusion-tensor imaging; SMS, Simultaneous multi-slice acquisition; TR, repetition time; TE, echo time; FoV, field of view.

Parameter	DTI based on ss-EPI	DTI based on SMS rs-EPI
TR	4,400 ms	2,800 ms
TE	77 ms	72 ms
Field of view	100 × 100 mm^2^	100 × 100 mm^2^
Voxel size	1.2 × 1.2 × 4.0 mm^3^	1.2 × 1.2 × 4.0 mm^3^
Slices and slice gap	36; 0	36; 0
Partial Fourier acquisition	6/8	6/8
GRAPPA acceleration factor	2	2
Slice acceleration factor	-	2
Gradient directions	20 (monopolar)	20 (monopolar)
Signal averages	b = 0 (5), b = 1,000 (2)	b = 0 (5), b = 1,000 (1)
k-space segments	1	5
Bandwidth	1,112 Hz/Px	714 Hz/Px
Echo spacing	1.0 ms	0.50 ms
Acquisition time	5:12 min	5:18 min

### 2.3 Assessment of image quality

Two independent readers (***initials blinded***; reader 1: clinical fellow with 5 years training in musculoskeletal radiology; reader 2: research fellow in musculoskeletal radiology with 2 years training in neuroradiology) rated on a 5-point-Likert-like scale (1, excellent; 2, good; 3, moderate; 4, poor; 5, non-diagnostic) the following qualitative imaging parameters: ghosting artifacts, anatomical distinction of the ulnar nerve and overall image quality.

### 2.4 Post-processing and measurements

All images were post-processed using the syngo.via platform (Version VB10A, Siemens Healthcare, Erlangen, Germany). For the DTI sequences, parametrical voxel-wise maps of the fractional anisotropy (FA) and mean diffusivity (MD) were automatically generated by the scanner's console (Table 2). For FA and MD measurements, both readers also independently placed standardized 6.4 mm^2^ regions of interest (ROIs) on the respective maps in the ulnar nerve at the level of the cubital tunnel. Potential partial-volume effects were avoided by defining the ROIs slightly smaller than the cross-sectional area of the nerve [12].

**Table 2:** Quantitative and qualitative image analysis: For both sequences and both readers quantitative parameters SNR, FA, MD are given as mean ± standard deviation (SD). Qualitative grading of artifacts, resolution and overall image quality is provided for each sequence and reader on a 5-point Likert scale (ghosting artifacts: 1, none; 2, low; 3, moderate; 4, high; 5, very high; overall image quality, anatomical depiction of ulnar nerve: 1, excellent; 2, good; 3, moderate; 4, poor; 5, non diagnostic). Paired t-tests were performed to assess potential between group differences. Corresponding p-values are given. Italics indicate statistical significance.

Reader		1		2		SMS rs-EPI vs. ss-EPI
Protocol		SMS rs-EPI	ss-EPI	SMS rs-EPI	ss-EPI	p-value
SNR		9.53	11.37	8.58	11.88	*0.002*
FA	Mean	0.578	0.631	0.607	0.631	0.11
	SD	±0.0961	±0.0755	±0.1174	±0.0848	
MD	Mean	1.27	1.32	1.42	1.41	0.93
	SD	±0.122	±0.184	±0.142	±0.211	
Overall image Quality		2.56	2.20	2.56	2.00	*0.008*
Anatomical depiction of ulnar nerve		2.22	1.80	2.44	1.70	*0.001*
Ghosting artifacts		2.11	2.30	1.89	2.10	0.4

### 2.5 SNR estimation

The SNR was estimated on the b0 images by dividing the signal of the ulnar nerve by the background noise (standard deviation of the signal) measured in an ROI at the edge of the field-of-view.

### 2.6 Statistical analysis

Statistical analyses were performed with SPSS (v20, IBM Corp., Somers, NY) and R (v. 3.2.4, R Foundation for Statistical Computing, Vienna, Austria). For FA and MD, a normal distribution was assumed and verified visually in the histogram. A paired Student's t-test was used for the comparison of FA and MD values between ss-EPI and SMS rs-EPI. Descriptive statistics of those were expressed as means and standard deviations. The reader ratings and SNR measurements were compared with an asymptotic Wilcoxon-Pratt signed-rank test for paired observations, and the data was described by median and interquartile range. A p-value <0.05 was considered significant, with Bonferroni correction for multiple comparisons where appropriate. Interreader agreement was measured with intraclass correlation coefficient (ICC) for FA and MD and with Cohen's kappa (**κ**) for the qualitative ratings. Respective confidence intervals were obtained via bootstrapping (R DescTools v. 0.99.5). Interreader agreement was deemed substantially different if the two compared values were outside of the respective other 95% confidence interval, as there is currently no widely accepted method of statistically computing a significance level when comparing interreader agreement [19]. The interreader agreement was interpreted as follows: slight agreement (<0.20), fair (0.20–0.39), moderate (0.40–0.59), substantial (0.60–0.79), or excellent (>0.80) agreement [20].

## 3 Results

### 3.1 Image acquisition

**Fig. 2:**
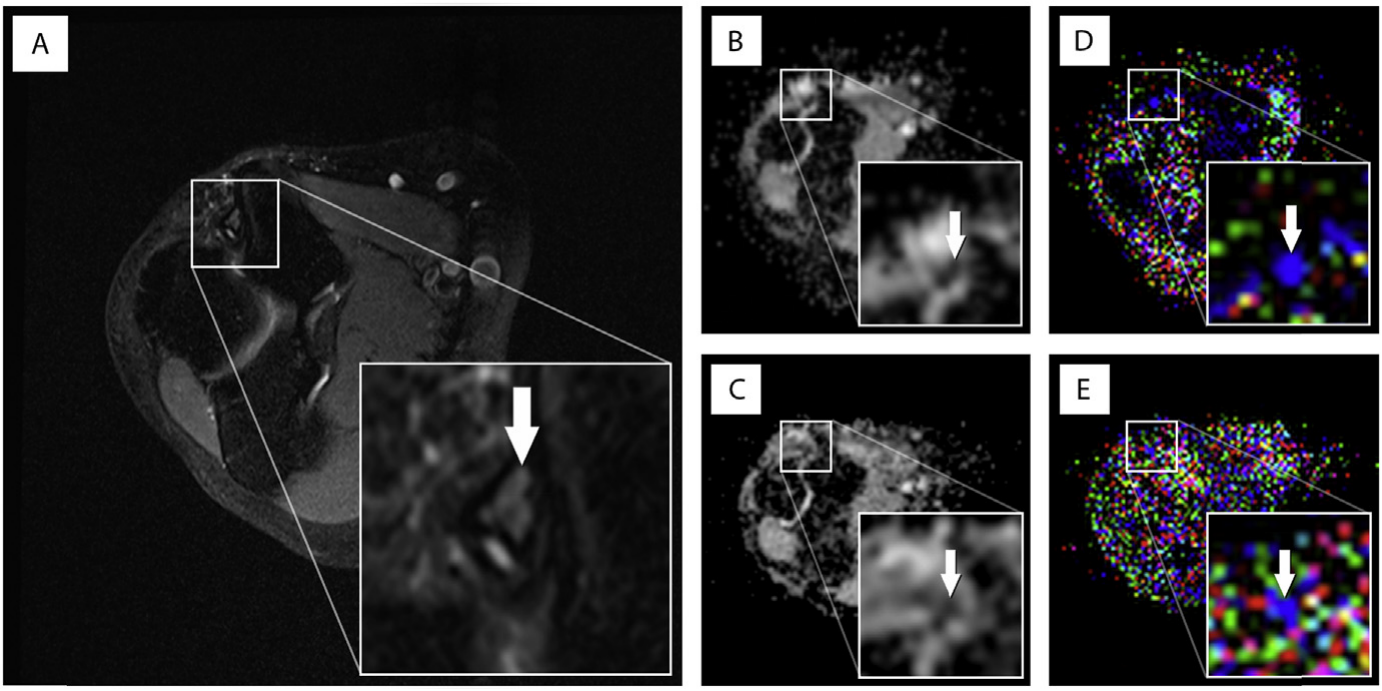
Comparison of anatomical depiction of the ulnar nerve and ghosting artifacts for a 27-year old female healthy volunteer. Panel (A) shows a T2-weighted, fat suppressed, axial image with delineation of the ulnar nerve in the cubital tunnel (white arrow) as standard of reference for anatomical correlation; panel (B), the automatically generated mean diffusivity (MD) map and (D) fractional anisotropy (FA) map for the ss-DTI at the same level, where anatomical depiction of the ulnar nerve was graded “good” and ghosting artefacts were rated “moderate” for both readers for both maps. Panel (C) and (E) demonstrates the corresponding rs-DTI maps. Here the MD map was graded “moderate” for anatomical depiction of the ulnar nerve by both readers and “moderate” for ghosting artifacts by reader 1 and “low” by reader 2. The FA map for rs-DTI was rated “poor” by both readers for anatomical depiction and “moderate” for ghosting artifacts for both readers.

All morphological and diffusion images were successfully acquired (for example FA and MD maps see Fig. 2). Specific absorption rate (SAR) limits were respected in all sequences without the need for switching to first-level mode.

### 3.2 Quantitative measurements

SNR measurements in SMS rs-DTI were 8.65 (range, 7.88–11.78) for reader 1 and with 8.53 (4.50–12.19) for reader 2 compared to ss-DTI with 10.62 (9.78–12.36) for reader 1 and with 10.24 (9.53–13.61) for reader 2 were not significantly different (rs-DTI p = 0.59, ss-DTI p = 0.33).

FA and MD values were similar for both readers (p = 0.65 and 0.15, respectively) and both sequences (p = 0.11 and 0.93, respectively). Inter-reader agreement of FA measurements was fair for both ss-EPI (ICC = 0.34; 95% confidence interval, −0.40 to 0.79) and for SMS rs-EPI (ICC = 0.34; −0.34 to 0.78). For MD measurements inter-reader agreement was moderate for conventional ss-DTI (ICC = 0.43; −0.12 to 0.81) and fair for SMS rs-EPI (ICC = 0.35; −0.31 to 0.79). Detailed diffusion metrics are given in Table 2.

### 3.3 Qualitative ratings

Anatomical depiction of the ulnar nerve and overall image quality were both perceived significantly better in the ss-EPI sequence (p = 0.002 and 0.008, respectively). Ghosting artifacts were not significantly different in the two sequences (p = 0.36). The interreader agreement was **κ** = 0.33 (0.10–0.77) for overall image quality, 0.37 (0.04–0.78) for and anatomical depiction 0.45 (0.04–0.86) for artifacts, corresponding to mostly fair to moderate agreement.

## 4 Discussion

### 4.1 The role of DTI for imaging of peripheral nerves

Until now diagnostic imaging of peripheral neuropathies with MR neurography rests upon prolonged T2-relaxation times in fat saturated pulse sequences to localize a intraneural edema within the course of the fascicles. However, T2 hyperintense signal alteration is not specific to any defined pathophysiological condition since they merely echo the grade of density and compartmentalization of water-hydrogen protons [21]. Another problem linked to imaging of peripheral nerves is the magic angle effect due to an artificial increase in T2-relaxation time [22]. This phenomenon is caused by interaction of water-hydrogen protons with the surrounding collagen matrix of the endoneurium and interfascicular compartments when nerve fibers run at an angle of 55° relative to the main static magnetic field [23]. Especially at the elbow the magic angle effect can limit selectivity between physiological pathologic conditions. Husarik et al. found that the T2w-signal of the ulnar nerve at the elbow is increased in about 60% of individuals without any symptoms of ulnar neuropathy [24].

Due to these limitations of T1 and T2 weighted anatomical MR imaging, DTI is a promising new approach to deliver more accurate diagnostic information about the nerve [25]. DTI relies on Brownian motion of water–hydrogen protons along different gradient directions. Changes in tissues self-diffusivity lead to alterations in derived DTI parametric maps of fractional anisotropy (FA) and mean diffusivity (MD). In the peripheral nervous system it is assumed that FA reflects the microstructural nerve integrity and mean diffusivity (MD) measures the density of the axonal membrane [26]. In general, peripheral nerves with their strong directionality seem to be predestinated for DTI. However, recent studies (mainly using ss-EPI) have indicated the technique may be limited in small nerves due to low in-plane resolution, wide slice thickness and partial volume contamination effects. Thus, in search of alternative sequences for DTI of peripheral nerves, in the present study the feasibility of SMS rs-EPI for DTI of the ulnar nerve at the cubital tunnel was tested and compared with conventional ss-EPI. We assessed quantitative data by means of FA, MD and SNR and qualitative data by means of ghosting and motion artifacts, anatomical distinction and overall image quality in axial DWI images in 10 healthy volunteers.

### 4.2 Technical considerations

We found that SMS rs-EPI yielded similar FA and MD as well as similar ghosting artifacts compared to ss-EPI, but significantly worse anatomical depiction of the ulnar nerve and poorer overall image quality, likely related to the slightly lower SNR. Still, obtained SNR values for the ulnar nerve in this study were in line with current literature for normal values in peripheral nerves [7, 9]. Sufficient SNR is important 1) in order to clearly identify the relatively small ulnar nerve at the elbow; 2) to minimize uncertainty of derived MD and FA values; and 3) to minimize uncertainty of the voxel-wise eigenvector of the diffusion tensor [27, 28].

At the same time, spatial resolution is a crucial factor for accurate DTI measurements. Within the cubital tunnel, the ulnar nerve normally is surrounded by fat, which poses an additional challenge to DTI because potential partial volume effects may significantly influence FA and MD measurements. To avoid this potential source of bias, we applied a relatively high spatial resolution, allowing us to draw ROIs small enough to avoid inclusion of extraneural fat, yet at the cost of lower SNR. In order to still maintain sufficient SNR, we chose the echo time as short as possible, used 6/8 partial Fourier sampling, applied 20 gradient directions and used a relatively low upper b-value of 1,000 s/mm^2^.

A study by Zhou et at. noted that SNR and spatial resolution had a strong impact on FA values in forearm nerves. They found FA values to be higher with low SNR and to be lower with higher voxel size [29]. Whilst keeping voxel size and ROIs as small as possible, both diffusion sequences seem to be appropriate for quantitative DTI. A reported phenomenon of SMS acquisition, which is not yet fully understood, is a potential increase of measured FA values at higher slice acceleration; however, in the present study we did not make such observations [30, 31]. In theory, given its shorter echo time and echo spacing, readout-segmented EPI inherently reduces susceptibility artifacts and geometric distortions compared to single-shot measurements. However, our visual image assessment did not reveal a significant advantage of SMS rs-EPI over ss-EPI. Recently another promising multi-shot DTI technique, termed multiplex sensitivity-encoding (MUSE) has been developed. This technique inherently corrects nonlinear shot-to-shot phase variations without using navigator echoes [32].

### 4.3 Limitations

We acknowledge several limitations of the current study: Our sample size with only ten healthy volunteers is relatively small, but in line with previous studies on peripheral nerves, and typical for pilot studies investigating novel techniques [9, 29]. Furthermore, we suppose that a larger sample size would not have had a relevant impact on the results of this study, because the inter-individual variability of DTI measurements was relatively low. Last, the phased-array coil used in our study comprised only four independent channels; hence, we could not take unlimited advantage of the multi-slice technique. However, previous studies have shown that a slice acceleration factor of 2 is the most reasonable compromise between image quality and acquisition time [7, 31].

## 5 Conclusion

In our study, we found that SMS rs-EPI was not superior to ss-EPI for DTI of the ulnar nerve at the elbow. In order to increase clinical acceptance and impact of diffusion-weighted imaging of peripheral nerves, further attempts are necessary to find the best technique.

## Declarations

### Author contribution statement

Michael Ho, Félix P. Kuhn, Lukas Filli: Conceived and designed the experiments; Performed the experiments; Wrote the paper.

Anton Becker: Performed the experiments; Wrote the paper.

Erika Ulbrich: Conceived and designed the experiments; Performed the experiments; Contributed reagents, materials, analysis tools or data.

Andrei Manoliu: Conceived and designed the experiments; Performed the experiments.

Matthias Eberhard: Contributed reagents, materials, analysis tools or data.

### Funding statement

This research did not receive any specific grant from funding agencies in the public, commercial, or not-for-profit sectors.

### Competing interest statement

The authors declare no conflict of interest.

### Additional information

No additional information is available for this paper.

## References

[1] Mondelli M., Giannini F., Ballerini M., Ginanneschi Martorelli E. (2005 Jul 15). Incidence of ulnar neuropathy at the elbow in the province of Siena (Italy). J. Neurol. Sci..

[2] Stewart J.D. (1987 Mar). The variable clinical manifestations of ulnar neuropathies at the elbow. J. Neurol. Neurosurg. Psychiatry.

[3] Breitenseher J.B., Kranz G., Hold A., Berzaczy D. (2015 Jul). MR neurography of ulnar nerve entrapment at the cubital tunnel: a diffusion tensor imaging study. Eur. Radiol..

[4] Baumer P., Dombert T., Staub F., Kaestel T. (2011 Jul). Ulnar neuropathy at the elbow: MR neurography-nerve T2 signal increase and caliber. Radiology.

[5] Filippou G., Mondelli M., Greco G., Bertoldi I. (2010 Jan-Feb). Ulnar neuropathy at the elbow: how frequent is the idiopathic form? An ultrasonographic study in a cohort of patients. Clin. Exp. Rheumatol..

[6] Jengojan S., Kovar, Breitenseher J., Weber M., Prayer D., Kasprian G. (2015 Jun). Acute radial nerve entrapment at the spiral groove: detection by DTI-based neurography. Eur. Radiol..

[7] Filli L., Piccirelli M., Kenkel D. (2016 Jun). Accelerated magnetic resonance diffusion tensor imaging of the median nerve using simultaneous multi-slice echo planar imaging with blipped CAIPIRINHA. Eur. Radiol..

[8] Basser P.J., Jones D.K. (2002 Nov–Dec). Diffusion-tensor MRI: theory, experimental design and data analysis - a technical review. NMR Biomed..

[9] Manoliu A., Ho M., Nanz D., Piccirelli M. (2016 Aug). Diffusion tensor imaging of lumbar nerve roots: comparison between fast readout-segmented and selective-excitation acquisitions. Invest. Radiol..

[10] Guggenberger R., Markovic D., Eppenberger P. (2012 Oct). Assessment of median nerve with MR neurography by using diffusion-tensor imaging: normative and pathologic diffusion values. Radiology.

[11] Takagi T., Nakamura M., Yamada M., Hikishima K. (2009 Feb 1). Visualization of peripheral nerve degeneration and regeneration: monitoring with diffusion tensor tractography. Neuroimage.

[12] Andreisek G., White L.M., Kassner A., Tomlinson G., Sussman M.S. (2009 Jan). Diffusion tensor imaging and fiber tractography of the median nerve at 1.5T: optimization of b value. Skeletal Radiol..

[13] Lindberg P.G., Feydy A., Le Viet D., Maier M.A., Drape J.L. (2013 Nov). Diffusion tensor imaging of the median nerve in recurrent carpal tunnel syndrome - initial experience. Eur. Radiol..

[14] Wadhwa V., Salaria S.N., Chhabra A. (2014 Jun 30). Granular cell tumor of the ulnar nerve: MR neurography characterization. J. Radiol. Case Rep..

[15] Baeumer P., Pham M., Ruetters M. (2014 Oct). Peripheral neuropathy: detection with diffusion-tensor imaging. Radiology.

[16] Porter D.A., Heidemann R.M. (2009 Aug). High resolution diffusion-weighted imaging using readout-segmented echo-planar imaging, parallel imaging and a two-dimensional navigator-based reacquisition. Magn. Reson. Med..

[17] Setsompop K., Gagoski B.A., Polimeni J.R., Witzel T., Wedeen V.J., Wald L.L. (2012 May). Blipped-controlled aliasing in parallel imaging for simultaneous multislice echo planar imaging with reduced g-factor penalty. Magn. Reson. Med..

[18] Frost R., Jezzard P., Douaud G., Clare S., Porter D.A., Miller K.L. (2015 Jul). Scan time reduction for readout-segmented EPI using simultaneous multislice acceleration: diffusion-weighted imaging at 3 and 7 Tesla. Magn. Reson. Med..

[19] Donner A., Shoukri Mm, Klar N., Bartfay E. (2000 Feb 15). Testing the equality of two dependent kappa statistics. Stat. Med..

[20] Landis J.R., Koch G.G. (1977). The measurement of observer agreement for categorical data. Biometrics.

[21] Pham M., Bäumer T., Bendszus M. (2014 Aug). Peripheral nerves and plexus: imaging by MR-neurography and high-resolution ultrasound. Curr. Opin. Neurol..

[22] Bowen B.C. (2004 Mar). Peripheral nerve imaging and the magic angle. AJNR Am J Neuroradiol.

[23] Bydder M., Rahal A., Fullerton Gd, Bydder G.M. (2007 Feb). The magic angle effect: a source of artifact, determinant of image contrast, and technique for imaging. J. Magn. Reson. Imag..

[24] Husarik D.B., Saupe N., Pfirrmann C.W.A., Jost B., Hodler J., Zanetti M. (2009 Jul). Elbow nerves: MR findings in 60 asymptomatic subjects--normal anatomy, variants, and pitfalls. Radiology.

[25] Breckwoldt M.O., Stock C., Xia A., Heckel A. (2015 Aug). Diffusion tensor imaging adds diagnostic accuracy in magnetic resonance neurography. Invest. Radiol..

[26] Alexander A.L., Hurley Sa, Samsonov Aa, Adluru N. (2011). Characterization of cerebral white matter properties using quantitative magnetic resonance imaging stains. Brain Connect..

[27] Polders D.L., Leemans A., Hendrikse J., Donahue M.J., Luijten P.R., Hoogduin J.M. (2011 Jun). Signal to noise ratio and uncertainty in diffusion tensor imaging at 1.5, 3.0, and 7.0 Tesla. J. Magn. Reson. Imag..

[28] Farrell J.A., Landman B.A., Jones C.K. (2007 Sep). Effects of signal-to-noise ratio on the accuracy and reproducibility of diffusion tensor imaging-derived fractional anisotropy, mean diffusivity, and principal eigenvector measurements at 1.5 T. J. Magn. Reson. Imag..

[29] Zhou Y., Kumaravel M., Patel V.S., Sheikh K.A., Narayana P.A. (2012 Oct). Diffusion tensor imaging of forearm nerves in humans. J. Magn. Reson. Imag..

[30] Lau A.Z., Tunnicliffe E.M., Frost R., Koopmans P.J., Tyler D.J., Robson M.D. (2015 Mar). Accelerated human cardiac diffusion tensor imaging using simultaneous multislice imaging. Magn. Reson. Med..

[31] Filli L., Piccirelli M., Kenkel D. (2015 Jul). Simultaneous multislice echo planar imaging with blipped controlled aliasing in parallel imaging results in higher acceleration: a promising technique for accelerated diffusion tensor imaging of skeletal muscle. Invest. Radiol..

[32] Chen N.K., Guidon A., Chang H.C. (2015 Jul). A robust multi-shot scan strategy for high-resolution diffusion weighted MRI enabled by multiplex sensitivity-encoding (MUSE). Invest. Radiol..

